# Serum Metabolomic Patterns in Patients With Aldosterone-Producing Adenoma

**DOI:** 10.3389/fmolb.2022.816469

**Published:** 2022-04-08

**Authors:** Yule Chen, Hanjiang Wang, Ke Wang, Guodong Zhu, Zhishang Yang, Min Wang, Wenbin Song

**Affiliations:** ^1^ Department of Urology, The First Affiliated Hospital of Xi’an Jiaotong University, Xi’an, China; ^2^ Oncology Research Laboratory, Key Laboratory of Environment and Genes Related to Diseases, Ministry of Education, Xi’an, China

**Keywords:** aldosterone-producing adenoma, metabolomics, serum, hypertension, adrenalectomy

## Abstract

Aldosterone-producing adenoma (APA), the main cause of endocrine hypertension, has recently been reported to be associated with other diseases, such as metabolic syndrome, but the detailed mechanism underlying this association remains unclear. Here, we used untargeted metabolomics and compared the abundance of serum metabolites between essential hypertension (EHT) and APA patients, as well as the serum metabolites of APA patients before and after adrenalectomy. Our results revealed 44 differential metabolites between APA and EHT patients and 39 differential metabolites between pre- and postoperative APA patients. Several metabolites involved in cardiovascular disease, obesity, and diabetes were dysregulated in APA patients compared to EHT patients, including arachidonic acid metabolites [e.g., 5(S)-HpETE and 12-HETE], amino acids (e.g., L-carnitine, taurine, and L-arginine), nucleotide metabolites (e.g., hypoxanthine) and cholesterol 3-sulfate. Importantly, the levels of hypoxanthine and cholesterol 3-sulfate, two metabolites that promote the development of atherosclerotic lesions and obesity, were originally increased in APA patients, but those elevated levels were reversed by adrenalectomy. Conversely, levels of L-carnitine and (3-carboxypropyl) trimethylammonium cation, two metabolites participating in lipid metabolism, were decreased in APA patients but increased postoperatively. We conclude that APA might participate in cardiovascular and metabolic diseases by regulating serum metabolites.

## Introduction

The renin-angiotensin-aldosterone system (RAAS) is an orchestrated hormonal cascade, that is, important for maintaining the homeostasis of fluids, electrolytes, and blood pressure; dysregulation of this cascade leads to cardiovascular disorders (Nappi and Sieg, 2011). Primary aldosteronism (PA), characterized as an overproduction of aldosterone under suppressed renin conditions (Gomez-Sanchez et al., 2020), is one of the most common causes of secondary hypertension, with a prevalence of 20% among patients with resistant hypertension (Byrd et al., 2018). Aldosterone-producing adenoma (APA) is one of the major subtypes of PA and can be cured by adrenalectomy (Funder, 2019). Elevated aldosterone results in low-renin hypertension, hypokalemia, and damage to target organs. It has been shown that patients with PA have a higher cardiovascular mortality than patients with essential hypertension (EHT), and this phenomenon was not explained by blood pressure because the patients were matched for cardiovascular risk (Reincke et al., 2012). In addition, PA-directed therapy reduced the excess morbidity (Loh and Sukor, 2020). Importantly, emerging evidence has indicated that patients with PA not only have an increased risk of cardiovascular events but also have increased risks of diabetes and metabolic syndrome (Monticone et al., 2018). Thus, aldosterone in PA patients might play a wide range of roles in the human body. However, the details of the underlying mechanism remain unclear.

In recent decades, much effort has been exerted to improve our understanding of PA. Metabolomics, a high-throughput approach, has been adopted to describe the metabolic features of PA using tumor or urinary samples from APA patients to reveal the characteristics or improve the diagnosis of PA (Spyroglou et al., 2021). However, serum is an ideal sample for disease diagnosis, disease monitoring, and mechanistic investigations and not only reflects the changes at the genomic and proteomic levels but also is influenced by environmental factors (Hashim et al., 2019). Thus, in this study, we preliminarily investigated the effects of APA on human disease using untargeted metabolomics of serum samples from EHT patients and APA patients before and after adrenalectomy.

## Materials and Methods

### Patients

We recruited a total of 11 patients with APA who had undergone unilateral retroperitoneal laparoscopic adrenalectomy in the Department of Urology at the First Affiliated Hospital of Xi’an Jiaotong University between January 2020 and May 2020. Serum renin and aldosterone were tested, and the aldosterone-renin ratio (ARR) was calculated to screen for APA. All patients had been diagnosed with unilateral cortical adenoma of the adrenal gland via enhanced computed tomography (CT). The diagnosis was confirmed by conducting an intravenous saline infusion test. Two of the patients aged <35 years were not recommended for adrenal vein sampling (AVS). The other patients were recommended for this procedure, but 3 patients refused it. Thus, in total, 6 patients underwent AVS, and the results were consistent with those of the CT scan. Eight patients had hypokalemia. No patients had abnormalities on cortisol or catecholamine tests. Three days after the adrenalectomy the serum aldosterone ARR and potassium levels were reexamined. We also recruited 9 patients with EHT admitted to the Department of Cardiovascular Medicine at the First Affiliated Hospital of Xi’an Jiaotong University as controls. EHT was defined as hypertension with no secondary hypertension cause, such as aberrant adrenal hormone levels, an adrenal mass, chronic kidney disease, renal artery stenosis, or hyperthyroidism. All patients agreed to participate in this study.

### Serum Collection

The EHT and preoperative APA patients were phlebotomized on the second day of hospitalization on an empty stomach. On the third day after adrenalectomy, the APA patients underwent a second blood draw. The peripheral blood sample was kept at 4°C for 2 h until it coagulated, followed by centrifugation at 2000 rpm for 10 min. The supernatant was collected and subjected to metabolomics analysis. One patient did not undergo postoperative phlebotomization because of a fever after adrenalectomy. Finally, 9 EHT samples and 11 preoperative APA samples were used to analyze the differential serum metabolites between EHT and APA patients. Additionally, 10 pairs of preoperative and postoperative samples were used to analyze the differential serum metabolites before and after adrenalectomy.

### Metabolite Extraction

To extract metabolites from the serum samples, 400 μL of cold extraction solvent methanol/acetonitrile/H_2_O (2:2:1, v/v/v) was added to 100 mg of sample and adequately vortexed. After vortexing, the samples were incubated on ice for 20 min and then centrifuged at 14,000 g for 20 min at 4°C. The supernatant was collected and dried in a vacuum centrifuge at 4°C. For LC–MS analysis, the samples were redissolved in 100 μL of acetonitrile/water (1:1, v/v) solvent and transferred to LC vials.

### LC–MS Analysis

For untargeted metabolomics of polar metabolites, extracts were analyzed using a quadrupole time-of-flight mass spectrometer (Sciex TripleTOF 6600) coupled to hydrophilic interaction chromatography via electrospray ionization (performed by Shanghai Applied Protein Technology Co., Ltd.). LC separation was performed on an ACQUIY UPLC BEH Amide column [2.1 mm × 100 mm, 1.7 µm particle size (Waters, Ireland) using a gradient] of solvent A (25 mM ammonium acetate and 25 mM ammonium hydroxide in water) to solvent B (acetonitrile). The gradient was 85% B for 1 min and was linearly reduced to 65% B over 11 min and then reduced to 40% over 0.1 min and kept at that level for 4 min; then, the gradient was increased to 85% over 0.1 min, and a 5-min re-equilibration period was employed. The flow rate was 0.4 ml/min, the column temperature was 25°C, the auto sampler temperature was 5°C, and the injection volume was 2 µL. The mass spectrometer was operated in both negative ion and positive ionization modes. The ESI source conditions were set as follows: ion source gas 1 (Gas 1) as 60, ion source gas 2 (gas 2) as 60, curtain gas (CUR) as 30, source temperature of 600°C, and IonSpray Voltage Floating (ISVF) of ± 5500 V. For MS acquisition, the instrument was set to acquire over the m/z range of 60–1000 Da, and the accumulation time for the TOF MS scan was set at 0.20 s/spectra. In auto MS/MS acquisition, the instrument was set to acquire over the m/z range of 25–1000 Da, and the accumulation time for the product ion scan was set at 0.05 s/spectra. The product ion scan was acquired using information-dependent acquisition (IDA) with the high-sensitivity mode selected. The parameters were set as follows: the collision energy (CE) was fixed at 35 V with ± 15 eV, a declustering potential (DP) was set at 60 V (+) and −60 V (−) to exclude isotopes within 4 Da, and the candidate ions to monitor per cycle were set at 10.

### Data Processing and Statistical Analysis

Processing of LC–MS data was carried out by Shanghai Applied Protein Technology Co., Ltd. The raw MS data (wiff.scan files) were converted to MzXML files using ProteoWizard MSConvert before being imported into freely available XCMS software. To select the peaks, the following parameters were used: centWave m/z = 25 ppm, peak width = c (10, 60), prefilter = c (10, 100). For peak grouping, bw = 5, mzwid = 0.025, and minfrac = 0.5 were used. Of the extracted ion features, only the variables with more than 50% of nonzero measurement values in at least one group were retained. Compound identification of metabolites by MS/MS spectra was conducted with an in-house database established with available authentic standards. After normalization to the total peak intensity, the processed data were uploaded before being imported into SIMCA-P (version 14.1, Umetrics, Umea, Sweden), where they were subjected to multivariate data analysis, including Pareto-scaled principal component analysis (PCA) and orthogonal partial least-squares discriminant analysis (OPLS-DA). Sevenfold cross-validation and response permutation testing were used to evaluate the robustness of the model. The variable importance in the projection (VIP) value of each variable in the OPLS-DA model was calculated to indicate its contribution to the classification. The normality of the distributions and the homogeneity of variances were checked using the Shapiro–Wilk test and Levene’s test, respectively. For comparisons between the EHT and APA groups, the p value of normally distributed samples was calculated using Student’s t-test (equal variances) or a two-tailed Welch’s t-test (unequal variances), and the *p* value of non-normally distributed samples was calculated using the Mann–Whitney U test. For comparisons between the preoperative and postoperative APA groups, the *p* value was calculated using a dependent t-test (normal distribution) or Wilcoxon signed-rank test (non-normal distribution). Metabolites with VIP >1 in OPLS‐DA analysis and *p* <0.05 were defined as significantly differential metabolites. Metabolites with VIP values >1 and 0.05 ≤ *p* <0.1 were defined as differential metabolites because the difference approached statistical significance (Carrillo et al., 2016). A Kyoto Encyclopedia of Genes and Genomes (KEGG) analysis was performed to evaluate the enrichment of metabolites in various pathways.

The baseline patient data (e.g., age, serum aldosterone) were analyzed using GraphPad Prism 8.0. Quantitative data are presented as the median and interquartile range. Differences in mean values between two groups were analyzed with the Wilcoxon test. The differences in count data between two groups were analyzed using the χ^2^ test.

## Results

### Clinical Characteristics of Participants

**FIGURE 1:**
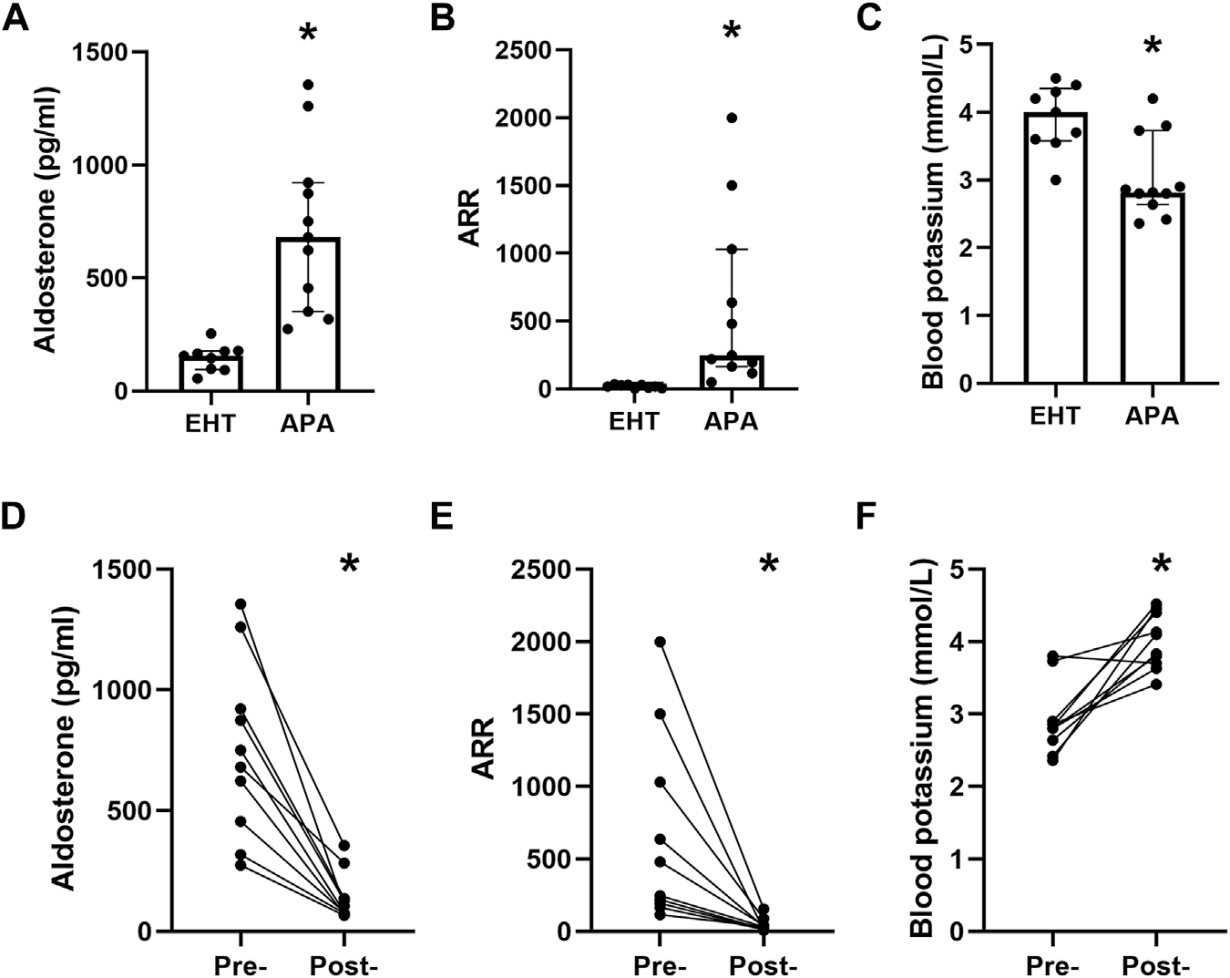
Baseline characteristics of the participants. **(A)** Serum aldosterone levels of the EHT and APA patients. **(B)** The aldosterone-to-renin ratio (ARR) of EHT and APA patients. **(C)** Serum levels of potassium in EHT and APA patients. **(D)** Preoperative and postoperative serum aldosterone levels. **(E)** Preoperative and postoperative serum ARRs. **(F)**. Preoperative and postoperative serum potassium levels. *p<0.05.

We did not find any significant difference in sex, body mass index (BMI), or blood pressure between the EHT and APA groups, though they did differ in age (Supplementary Table S1). The serum aldosterone levels and ARR in the APA group were significantly higher than those in the EHT group (Figures 1A,B). The level of serum potassium in the APA group was significantly lower than that in the EHT group (Figure 1C). Three days after adrenalectomy, serum aldosterone levels and ARR were significantly decreased, and all patients with hypokalemia had normal potassium levels (Figures 1D–F).

### Differential Metabolites Between EHT and APA Patients

The PCA indicated good clustering of the QC group, and no extreme outliers were observed (Supplementary Figure S1A). The OPLS-DA analysis indicated clear separation between the EHT and APA groups in both positive and negative ion modes (Supplementary Figure S1B). The results of the permutation test strongly indicated that original models in both positive (R2 = 0.6675, Q2 =−0.3839) and negative (R2Y = 0.9786, Q2 = −0.1739) ion modes were valid (Supplementary Figure S1C).

**FIGURE 2:**
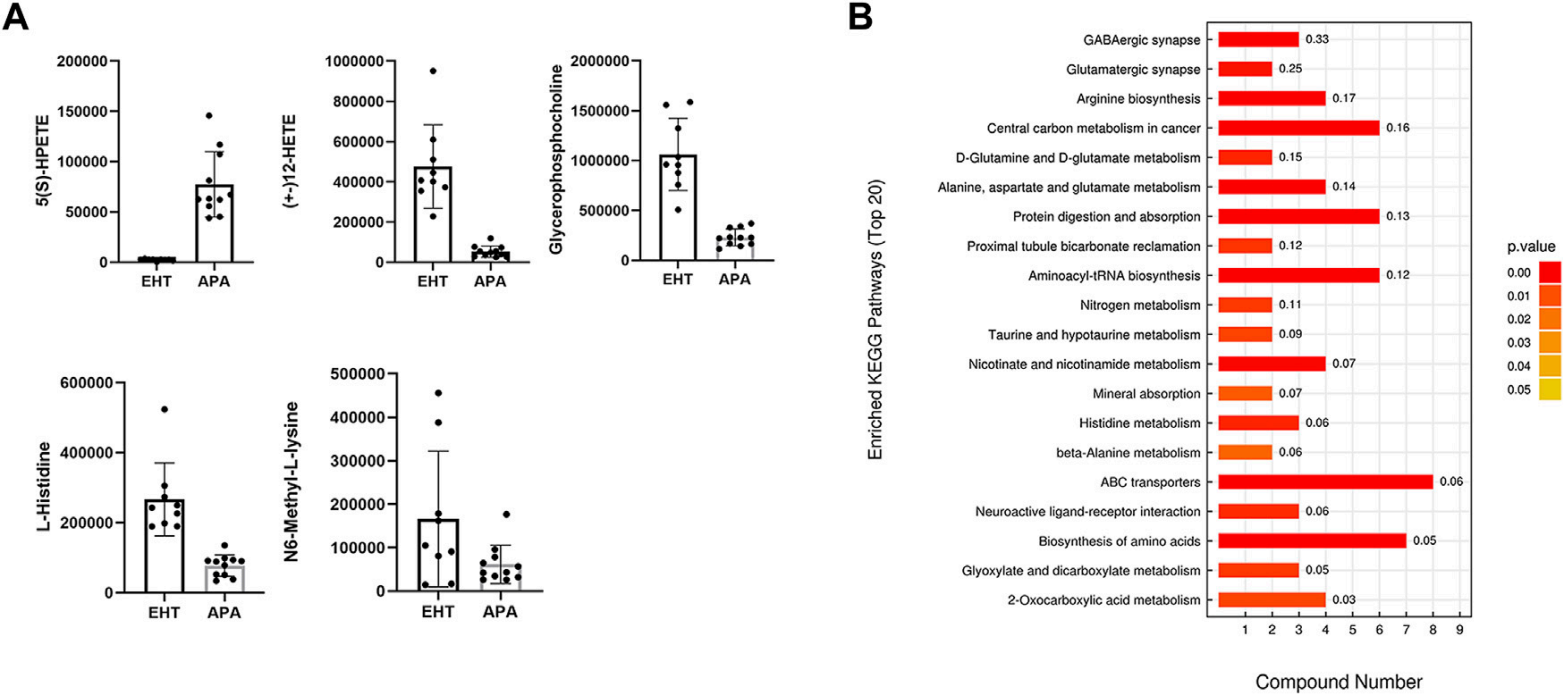
Differential metabolites between EHT and APA patients. **(A)** Top five differential serum metabolites detected by LC–MS analysis in EHT and APA patients. **(B)** KEGG analysis of the differential metabolites detected by LC–MS analysis in EHT and APA patients.

In our study, a total of 164 and 168 known biochemical compounds were identified by LC–MS analysis in positive ion and negative ion modes, respectively. Based on our selection criteria, we detected 22 significantly differential metabolites between the EHT and APA groups (VIP >1, *p* <0.05) and one differential metabolite (VIP >1, 0.05 ≤ *p* <0.1) in the positive ion mode, and we identified 14 significantly differential metabolites (VIP >1, p <0.05) and 8 differential metabolites (VIP >1, 0.05 ≤ *p* <0.1) between the EHT and APA groups in the negative ion mode. All significantly differential metabolites and differential metabolites are listed in Table 1. Combining the results from both positive and negative ion modes revealed that 10 metabolites exhibited higher abundance in the APA group, while 34 metabolites exhibited lower abundance in the APA group than in the EHT group. Among the metabolites abundant in the APA group, 5(S)-HpETE showed the largest fold change and was 32-fold higher in the APA group than in the EHT group. Among the metabolites abundant in the EHT group, 12-HETE showed the largest fold change and was 9-fold higher in the EHT group than in the APA group (Figure 2A, Table 1).

**TABLE 1:** Differential metabolites between APA and EHT patients.

Positive ion mode				Negative ion mode			
Name	VIP	Fold change (APA vs EHT)	*p* value	Name	VIP	Fold change (APA vs EHT)	*p* value
**Glycerophosphocholine**	4.39	0.22	0.00	**(+-)12-HETE**	7.36	0.11	0.00
**L-Histidine**	2.13	0.29	0.00	**L-Glutamate**	5.55	0.41	0.00
**N6-Methyl-L-lysine**	1.34	0.37	0.05	**Succinate**	1.36	0.52	0.00
**Trigonelline**	1.18	0.45	0.01	**L-Aspartate**	1.77	0.59	0.00
**D-Proline**	3.60	0.48	0.00	**1-Oleoyl-L-.alpha.-lysophosphatidic acid**	1.31	0.59	0.01
**L-Glutamate**	1.53	0.50	0.00	**Hydroxyisocaproic acid**	2.36	0.64	0.02
**Betaine**	2.73	0.51	0.00	**D-Quinovose**	1.88	0.66	0.05
**Ornithine**	1.21	0.53	0.01	**2-Hydroxy-3-methylbutyric acid**	2.96	0.66	0.05
**2-Methylbutyroylcarnitine**	1.64	0.55	0.02	**Ammelide**	1.17	0.81	0.00
**L-Lysine**	1.36	0.55	0.03	**Hypoxanthine**	7.53	1.49	0.02
**L-Glutamine**	3.60	0.62	0.00	**3-Hydroxycapric acid**	2.70	1.52	0.02
**1-Stearoyl-2-hydroxy-sn-glycero-3-phosphocholine**	1.80	0.62	0.01	**Bisindolylmaleimide I**	2.82	2.35	0.03
**(3-Carboxypropyl) trimethylammonium cation**	2.59	0.62	0.00	**pregnenolone sulfate**	4.36	2.38	0.04
**L-Citrulline**	1.06	0.66	0.02	**5(S)-HpETE**	2.95	32.38	0.00
**L-Leucine**	2.07	0.68	0.02	Phenol	6.98	0.51	0.06
**1-Oleoyl-sn-glycero-3-phosphocholine**	5.60	0.68	0.05	D-Sorbitol	1.14	0.57	0.08
**Nicotinamide**	1.56	0.73	0.04	L-Iditol	2.14	0.65	0.05
**L-Carnitine**	10.97	0.77	0.00	Capric acid	1.28	0.75	0.07
**Taurine**	1.75	0.83	0.02	L-Proline	2.01	0.81	0.09
**Sphingomyelin (d18:1/18:0)**	2.22	1.34	0.02	L-Glutamine	1.10	0.87	0.09
**Hypoxanthine**	2.26	1.55	0.02	2-Oxoadipic acid	8.58	1.03	0.08
**Thioetheramide-PC**	1.03	1.56	0.04	Cholesterol 3-sulfate	8.38	1.39	0.07
L-Arginine	4.99	0.57	0.05				

All significantly differential metabolites (VIP >1, p < 0.05) are shown in bold and differential metabolites (VIP >1, 0.05 ≤ *p* < 0.1) are shown in regular font.

Bold values represents the significant differential metabolites.

A KEGG enrichment analysis was conducted on all significantly differential metabolites detected by both positive and negative ion modes. The results indicated that the significantly differential metabolites were enriched in several pathways, such as those involving GABAergic synapses, arginine synthesis, and ABC transporters (Figure 2B).

### Differential Metabolites Between the Preoperative and Postoperative Serum of APA Patients

The PCA indicated good clustering of the QC group, and no extreme outliers were observed (Supplementary Figure S2A). The OPLS-DA analysis indicated clear separation between the preoperative and postoperative groups, in both positive and negative ion modes (Supplementary Figure S2B). The results of the permutation test strongly indicated that the original models of both positive (R2 = 0.9196, Q2 =−0.2122) and negative (R2Y = 0.8224, Q2 = −0.3506) ion modes were valid (Supplementary Figure S2C).

Based on our selection criteria, we detected 17 significantly differential metabolites (VIP >1, p <0.05) and 6 differential metabolites (VIP >1, 0.05 ≤ *p* <0.1) between the preoperative and postoperative groups in the positive ion mode, and we identified 14 significantly differential metabolites (VIP >1, *p* <0.05) and 3 differential metabolites (VIP >1, 0.05 ≤ *p* < 0.1) between the preoperative and postoperative groups in the negative ion mode. All significantly differential metabolites and differential metabolites are listed in Table 2.

**TABLE 2:** Differential metabolites between preoperative and postoperative APA patients.

Positive ion mode				Negative ion mode			
Name	VIP	Fold change (postoperative vs. preoperative)	*p* value	Name	VIP	Fold change (postoperative vs. preoperative)	*p* value
**Trimethylamine N-oxide**	1.70	0.32	0.04	**Pyrocatechol**	1.95	0.08	0.00
**Trigonelline**	2.21	0.33	0.00	**3-Indolepropionic acid**	2.68	0.18	0.02
**Nicotinamide**	3.71	0.38	0.00	**Indoxyl sulfate**	10.53	0.48	0.03
**L-Lysine**	2.18	0.48	0.01	**Hypoxanthine**	9.58	0.55	0.00
**Allopurinol riboside**	2.86	0.50	0.01	**Cholesterol 3-sulfate**	13.22	0.60	0.00
**Inosine**	5.45	0.53	0.01	**1-Palmitoyl-2-hydroxy-sn-glycero-3-phosphoethanolamine**	1.34	0.76	0.01
**Uracil**	1.09	0.60	0.00	**Taurine**	1.90	1.25	0.05
**Hypoxanthine**	15.68	0.63	0.02	**D-Mannose**	1.24	1.50	0.01
**Phthalic acid Mono-2-ethylhexyl Ester**	1.70	0.64	0.03	**D-Lyxose**	1.62	1.57	0.01
**Glycerophosphocholine**	3.58	0.65	0.03	**Methylmalonic acid**	1.45	1.77	0.00
**L-Citrulline**	1.36	0.66	0.01	**D-Threitol**	1.02	1.98	0.00
**1-Palmitoyllysophosphatidylcholine**	1.23	0.67	0.01	**myo-Inositol**	1.33	2.15	0.02
**1-Palmitoyl-2-hydroxy-sn-glycero-3-phosphoethanolamine**	1.66	0.74	0.01	**Acetoacetic acid**	1.33	2.21	0.00
**1-Stearoyl-2-oleoyl-sn-glycerol 3-phosphocholine (SOPC)**	13.56	1.24	0.05	**D-Maltose**	1.28	8.55	0.00
**L-Carnitine**	16.64	1.40	0.03	Chenodeoxycholate	1.49	0.31	0.07
**Phe-Ile**	1.26	2.04	0.01	Allantoin	1.97	0.84	0.07
**Cellobiose**	1.99	7.37	0.00	DL-3-Phenyllactic acid	1.32	1.66	0.06
N1-Methyl-2-pyridone-5-carboxamide	3.32	0.50	0.06				
Decanoyl-L-carnitine	2.99	0.64	0.09				
1-Oleoyl-sn-glycero-3-phosphocholine	8.81	0.72	0.05				
1-Palmitoyl-sn-glycero-3-phosphocholine	2.38	0.81	0.08				
Betaine	1.75	0.83	0.08				
(3-Carboxypropyl) trimethylammonium cation	3.34	1.61	0.06				

All significantly differential metabolites (VIP >1, p <0.05) are shown in bold and differential metabolites (VIP >1, 0.05 ≤ *p* <0.1) are shown in regular font.

Bold values represents the significant differential metabolites.

**FIGURE 3:**
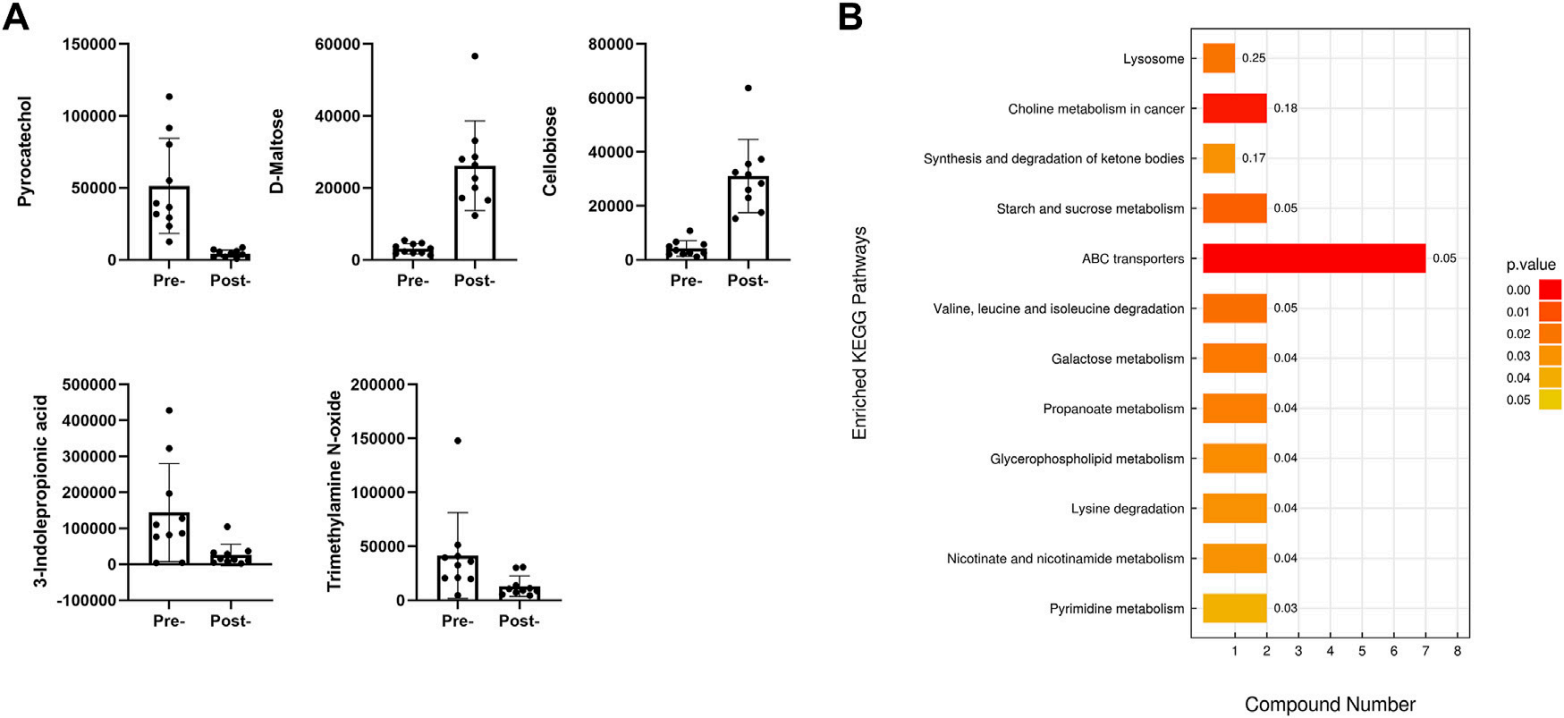
Differential serum metabolites between preoperative and postoperative APA patients. **(A)** Top five differential serum metabolites detected by LC–MS analysis between preoperative and postoperative APA patients. **(B)** KEGG analysis of the differential serum metabolites detected by LC–MS analysis between preoperative and postoperative APA patients.

Combining the results of both positive and negative ion modes revealed that 10 metabolites exhibited higher abundance in the preoperative APA group, while 34 metabolites exhibited higher abundance in the postoperative APA group. Among the metabolites abundant in the preoperative APA group, cellobiose showed the largest fold change and was 8.5-fold higher in the preoperative APA group than in the postoperative APA group. Among the metabolites abundant in the postoperative APA group, pyrocatechol showed the largest fold change and was 12.5-fold higher in the postoperative APA group than in the preoperative APA group (Figure 3A).

A KEGG enrichment analysis was conducted including all significantly differential metabolites detected by both positive and negative ion modes. The results indicated that the significantly differential metabolites were enriched in 12 pathways, such as those involving ABC transporters and lysine degradation (Figure 3B).

### APA-Regulated Serum Metabolites

**FIGURE 4:**
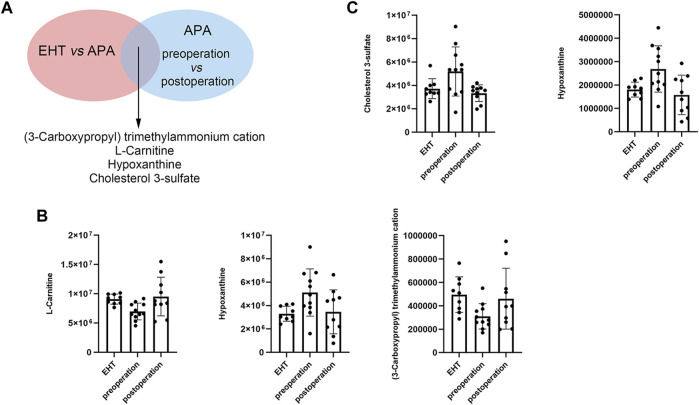
APA-regulated serum metabolites. **(A)** Outline of the analysis. **(B)** Abundance of metabolites that might be regulated by APA identified in positive ion modes. **(C)** Abundance of metabolites that might be regulated by APA identified in negative ion modes.

Next, we conducted a conjoint analysis by combining the significantly differential metabolites and differential metabolites between the serum of the EHT and APA groups and those between the serum of the preoperative and postoperative APA patients (Figure 4A). The results indicated that hypoxanthine and cholesterol 3-sulfate levels were increased in APA patients compared with those in EHT patients and were decreased postoperatively (Figures 4B,C). Conversely, L-carnitine and (3-carboxypropyl) trimethylammonium cation levels were decreased in APA patients compared with those in EHT patients and were increased postoperatively (Figures 4B,C). In particular, a change in hypoxanthine levels was detected in both positive and negative ion modes (Figures 4B,C).

## Discussion

Early studies on PA metabolomics mainly focused on steroid profiling, which indicated elevated 18-hydroxycortisol and 18-oxocortisol levels in APA patients (Spyroglou et al., 2021). Recently, Alessandro Lana et al. performed metabolic profiling to distinguish between EHT and APA patients and between APA and bilateral adrenal hyperplasia (BAH) patients.

They found that purine nucleosides and the related catabolites deoxyadenosine and uric acid were considerably different between EHT and PA patients; however, the adenosine deamination catabolites (deoxyinosine, hypoxanthine, and IMP), free amino acids (histidine and taurine), and the pyrimidine diphosphate CDP exhibited higher discriminatory power when comparing APA and BAH groups in a sex-independent manner (Lana et al., 2019). Another study focusing on distinct signatures of KCNJ5 and CACNA1D mutant APAs was performed by Murakami and colleagues. They revealed that purine metabolism is activated in KCNJ5 mutant APA (Murakami et al., 2019).

In the current study, we compared the abundance of serum metabolites in EHT and APA patients using untargeted metabolomics. The results revealed 34 decreased and 10 increased metabolites in APA patients compared with EHT patients. KEGG analysis of the differential metabolites between APA and EHT patients found that they were enriched in several pathways involved in central carbon metabolism, amino acid metabolism, and ABC transporters, indicating that APA might have a broad effect on human metabolism. Among these metabolites, we observed differences in two arachidonic acid catabolites, 5(S)-HpETE and 12-HETE, between EHT and APA patients. Since arachidonic acid is widely involved in inflammation, obesity, diabetes, hypercholesterolemia, and cardiovascular disease, it is possible that APA facilitates cardiovascular and metabolic diseases via changes in arachidonic acid metabolism (Sonnweber et al., 2018). Furthermore, both 5(S)-HpETE and 12-HETE levels showed clear separation between APA and EHT patients, indicating that these metabolites might be used as biomarkers to screen for APA.

We also found that APA patients had lower levels of several amino acids (e.g., L-histidine, D-proline, L-glutamate, ornithine, L-lysine, L-glutamine, L-citrulline, L-leucine, L-carnitine, taurine, L-arginine, L-aspartate) than those of EHT patients; some of these amino acids have been reported to play a protective role against cardiovascular diseases. For example, L-arginine was found to improve artery diameter and endothelial function in humans and improve endothelial function and reduce atherosclerotic plaques in animal models (Zaric et al., 2020), while taurine supplementation improved left ventricular function and reduced atherosclerotic lesion formation (Zaric et al., 2020). Thus, amino acid metabolism might be another mechanism underlying APA-mediated cardiovascular risks.

We also compared serum metabolites in preoperative and postoperative patients with APA. The results indicated 25 downregulated and 14 upregulated metabolites after the operation. KEGG analysis of the differential metabolites showed that they were enriched in pathways involved in ABC transporters, pyrimidine metabolism, and lysine degradation. Using a conjoint analysis that combined the differential serum metabolites of the EHT and APA groups and of the preoperative and postoperative APA patients, we found that hypoxanthine and cholesterol 3-sulfate levels were increased in APA patients compared with those in EHT patients but decreased postoperatively. Conversely, L-carnitine and (3-carboxypropyl) trimethylammonium cation levels were decreased in APA patients compared with those in EHT patients but increased postoperatively. Most of these metabolites have been found to be associated with metabolic syndrome or cardiovascular diseases. For example, cholesterol 3-sulfate is present on a variety of cells and in human low-density lipoproteins and is believed to participate in platelet adhesion and atherosclerotic lesions (Merten et al., 2001). Hypoxanthine is a product of purine metabolism and is associated with obesity and smoking (Furuhashi et al., 2020). L-carnitine transports long-chain fatty acids into the mitochondrial matrix, thus allowing the cells to degrade fat (Pekala et al., 2011). L-carnitine supplementation thus provides a protective effect against overweight (Askarpour et al., 2020) and cardiovascular events (Wang et al., 2018). (3-Carboxypropyl) trimethylammonium is derived from betaine, a dietary compound that participates in lipid metabolism (Airaksinen et al., 2018). However, we did not observe any changes in levels of 5(S)-HpETE or 12-HETE, possibly due to the short interval (only 3 d) between the adrenalectomy and postoperative blood sampling.

The main limitation of our study was its small sample size. Further studies with larger sample sizes are needed to validate the differential metabolites and to investigate their clinical significance. In addition, postoperative serum samples were collected only 3 d after adrenalectomy. This might miss potential metabolites regulated by APA. Thus, longer follow-up times are also needed in future studies. Finally, the age difference between APA and EHT patients might affect the accuracy of the results.

## Conclusion

In this study, we identified differential serum metabolites between patients with EHT and APA, as well as differential serum metabolites between APA patients pre- and post-adrenalectomy. We found that several metabolites involved in cardiovascular diseases, obesity, and diabetes were dysregulated in APA patients compared to those in EHT patients. In particular, APA patients had higher hypoxanthine and cholesterol 3-sulfate levels but lower L-carnitine and (3-carboxypropyl) trimethylammonium cations levels than EHT patients. The abnormality of the concentrations of these metabolites was reversed by adrenalectomy. We conclude that APA might influence cardiovascular and metabolic diseases by regulating these metabolites.

## Data Availability

The original contributions presented in the study are included in the article/Supplementary Material, further inquiries can be directed to the corresponding author.

## Supplementary Material

Supplementary Figure S1 PCA and OPLS-DA score plots of EHT and APA patients. **(A)** PCA score plot of the serum of EHT and APA patients. **(B)** OPLS-DA score plot of the serum of EHT and APA patients. **(C)** Permutation plots for the OPLSDA model showing R2 and Q2 values.

Supplementary Figure S2 PCA and OPLS-DA score plots of preoperative and postoperative APA patients. **(A)** PCA score plot of the serum of preoperative and postoperative APA patients. **(B)** OPLS-DA score plot of the serum of preoperative and postoperative APA patients. **(C)** Permutation plots for the OPLS-DA model showing R2 and Q2 values.

Supplementary Table S1 Baseline characteristics of the participants.
