# Supplementary material for: Serum Metabolomic Patterns in Patients With Aldosterone-Producing Adenoma
Source: Front Mol Biosci. 2022 Apr 8;9:816469. doi: 10.3389/fmolb.2022.816469 (PMC9023800; doi:10.3389/fmolb.2022.816469)
Supplement: Supplementary file 1 [file Table1.pdf]

Supplementary Table 1. Baseline characteristics of the participants

	EHT (n=9)	APA (n=11)	p value
Age (years)	49 (14)	44 (14)	**0.04**
SBP (mmHg)	154 (35.5)	147 (19)	0.95
DBP (mmHg)	85 (20)	98 (14)	0.08
BMI （kg/m^2）	24.5 (5.35)	24 (2.2)	0.56
Sex			＞0.99
Male	5	5	
Female	4	6	

Legend: SBP-systolic blood pressure, DBP-diastolic blood pressure, BMI-body mass index.
