# Supplementary figures and images for: Serum Metabolomic Patterns in Patients With Aldosterone-Producing Adenoma
Source: Front Mol Biosci. 2022 Apr 8;9:816469. doi: 10.3389/fmolb.2022.816469 (PMC9023800; doi:10.3389/fmolb.2022.816469)

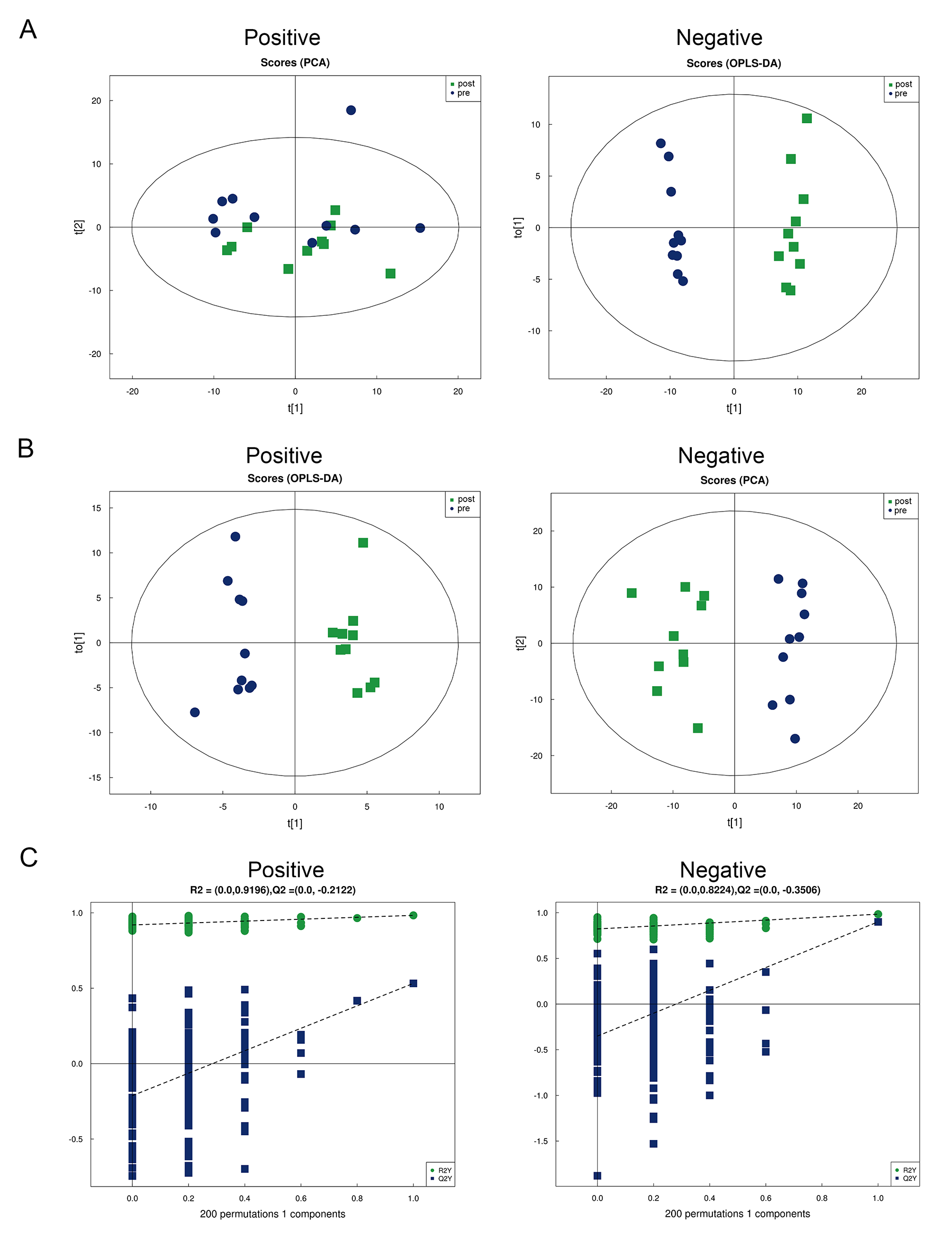

Supplement: Supplementary file 2 [file Image2.tif]

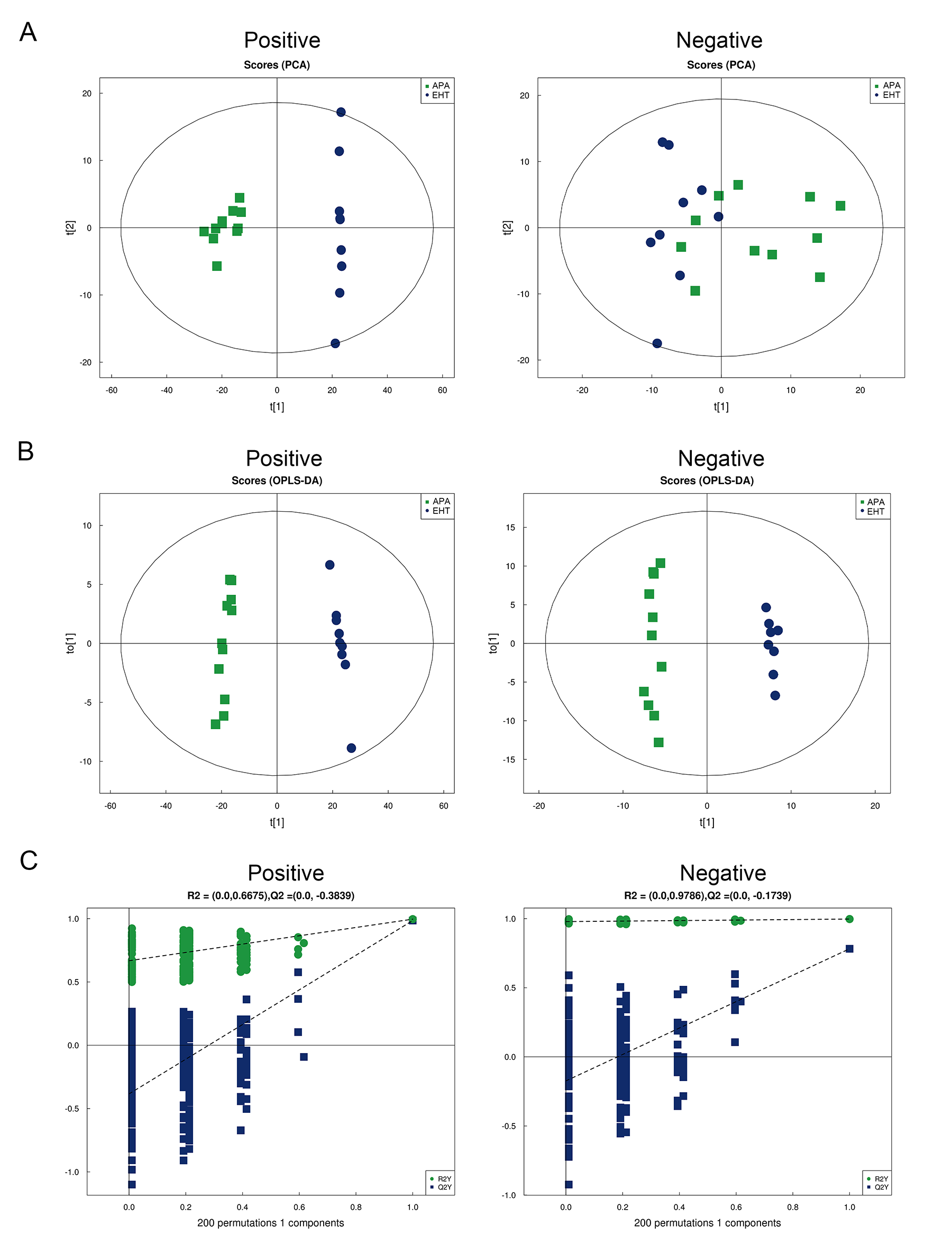

Supplement: Supplementary file 3 [file Image1.tif]
